# Investigation of Dose Minimisation Protocol for ^18^F-FDG PET-CT in the Management of Lymphoma Postchemotherapy Followup

**DOI:** 10.1100/2012/208135

**Published:** 2012-04-01

**Authors:** L. I. Sonoda, B. Sanghera, W. L. Wong

**Affiliations:** Paul Strickland Scanner Centre, Mount Vernon Hospital, Northwood, Middlesex HA6 2RN, UK

## Abstract

*Introduction*. ^18^F-FDG-PET-CT plays an important role in the management of lymphoma postchemotherapy followup. Some centres perform prechemotherapy baseline CT and postchemotherapy PETCT. With a concern of radiation burden, especially in young patients, this study aimed to assess if PETCT radiation dose could be reduced. *Methods*. Retrospective analysis of 100 lymphoma patients was performed to record sites of disease on prechemotherapy CT and postchemotherapy PETCT. The potential reduction in radiation and time achieved with PETCT limited to sites of known disease identified on prechemotherapy CT was calculated. *Results*. No FDG-uptake was seen in 72 cases. FDG uptake at known disease sites was seen in 24. Of the remaining 4, one had clinically significant pathology, a rectal adenocarcinoma. PETCT did not reveal any unexpected sites of lymphoma. Limiting PETCT to sites of known disease would have saved a mean radiation dose of 4 mSv (27.3%), with a mean time of 16 minutes. *Conclusion*. Our study suggests that young patients may benefit from reduced radiation by limiting PETCT to sites of known disease with low risk of missing significant pathology. However, in older patients, with increased incidence of asymptomatic synchronous malignancies, whole-body PETCT is advisable unless prechemotherapy PETCT has been performed.

## 1. Introduction

^18^F-Fluro-deoxyglucose positron emission tomography and computed tomography (PET-CT) is a powerful imaging tool for assessing response to chemotherapy in lymphoma patients. PETCT can accurately distinguish residual disease from sequelae of treatment in postchemotherapy lesions [1–13]. However, many lymphoma patients are young, and repeated exposure to ionising radiation during their patient journey can lead to secondary cancers in later life [14]. It is widely acknowledged that radiation dose should be kept as low as reasonably practicable, whilst ensuring imaging protocols are optimised to ensure efficacy of diagnostic information [15, 16]. This study aims to assess whether PETCT from skull base to pubic symphysis is feasible for assessing response to chemotherapy in lymphoma patients, and to calculate possible radiation dose and scan-time reductions achievable with a limited PETCT protocol.

## 2. Materials and Methods

### 2.1. Database

Local ethical board approval was obtained for review and publication of data. Our cohort consisting of 100 subjects (55 males, 45 females) had an age range of 9 to 78 years with a mean of 41.7 years (56 patients were 45 years or younger). 50 patients were identified with Hodgkin's lymphoma (HL) and 50 patients with non-Hodgkin's lymphoma (NHL). The distribution of the disease sites on the prechemotherapy CT studies is shown in Table 1.

The chemotherapy regime varied depending on the type of lymphoma. 83 of the 100 patients (83%) had received 6 cycles or more of chemotherapy prior to PETCT. All 100 patients (100%) had been assessed prior to chemotherapy with diagnostic CT. Most CTs were performed at hospitals remote from our institution and reported by consultant radiologists at those hospitals. Reports for these CTs were available for review. Sites of disease on prechemotherapy CT were grouped into four categories: (A) disease in the neck (including supraclavicular fossae, SCF), (B) disease in the chest (including axillae), (C) disease in the abdomen, and (D) disease in the pelvis.

### 2.2. PETCT

All patients were imaged with a discovery ST (GE Medical Systems, Waukesha, Wisconsin) PETCT postchemotherapy using our standard whole-body scan protocol. The PET enabled simultaneous acquisition of 47 transverse PET images per field of view with intersection spacing of 3.27 mm. The field of view and pixel size of the PET images reconstructed for fusion were 60 cm and 4.7, mm respectively, with a matrix size of 128 by 128. The scanner included a four-detector row spiral CT with the following routine imaging parameters: detector row configuration 4 × 2.5 mm, pitch 1.5, table speed 15 mm per gantry rotation, and rotation time 0.8 sec.

After a six-hour fasting period, patients were injected with 4.5 MBq/kg body weight of FDG. Blood glucose levels were checked before injection. Following 60 ± 5 minutes of uptake period, time during which the patient was instructed to rest without talking or chewing, data was acquired. CT was performed from skull base to pelvis by performing a scout view using 10 mA and 120 kVp scanning parameters, followed by a spiral CT with 80 mA, 140 kVp. No intravenous contrast was administered, and water was used to delineate bowel. On completion of CT, 2D PET emission data (4 minutes per bed position covering an axial FOV of 15.7 cm with a 3-slice overlap) was obtained. The total acquisition time varied between 25 and 30 minutes per patient. CT data was used for attenuation correction. Data was displayed on a workstation (Xeleris, GE Medical Systems) for analysis.

### 2.3. Image Interpretation and Data Analysis

PETCT studies were visually assessed on the manufacturer's proprietary viewing workstations by one experienced observer trained in diagnostic radiology and nuclear medicine and with 20 years experience of PET (WLW). All postchemotherapy PETCT reports were reviewed by one investigator (TM), unaware of other imaging/clinical findings or final outcome and any sites of possible disease not already identified on the prechemotherapy CT were recorded. All patients with unexpected sites of FDG uptake were sent questionnaires to obtain relevant patient information that included subsequent investigations, final outcome, and period of followup. Case notes were reviewed, and when relevant, the prechemotherapy CTs were reviewed.

### 2.4. Radiation Dose

The total effective dose to the patient was calculated, incorporating both PET and CT contributions from the postchemotherapy PETCT scan. In the case of PET, absorbed doses per unit activity of ^18^F-FDG administered to organs or tissues are as reported in Publication 80 of the International Commission on Radiological Protection (ICRP) [17]. Publication 80 of the ICRP uses tissue-weighting factors to derive effective doses per unit-injected activity that are sensitive to the subject's age. In the case of CT, the widely used ImPACT patient dosimetry software calculator (version 0.99s) was used to calculate the CT dose for each individual patient [18]. To optimise results, patient-specific details and scanner-specific parameters were considered in the calculations. Furthermore, we modified the tube current setting on the dose calculator from 80 mA to 120 mA for patients over 100 kg to reflect our routine clinical protocols and to facilitate more accurate results in obese patients. In each subject, the effective dose from the actual PETCT scan and the effective dose if only known disease sites had been imaged on the PETCT study were calculated.

## 3. Results

On review of PETCT reports, 72 patients had no FDG uptake to suggest residual disease. Twenty-four patients had FDG uptake consistent with active disease on PETCT in areas where disease was seen on prechemotherapy CT. Four patients were identified to have FDG uptake in areas where disease had not previously been identified on prechemotherapy CT. Two of these subjects presented with mild FDG uptake in the paranasal sinuses, confirmed as sinusitis on followup. In one patient, a 9 mm pulmonary nodule with a maximum standardised uptake value (SUVmax) of 3.1 was observed. Review of the prechemotherapy CT revealed that the nodule was present but had not been reported. The nodule remained unchanged in size over the course of the following year. In the fourth case, the scan of a 55-year-old man highlighted a small area of intense FDG uptake in the rectum, proven by sigmoidoscopy and biopsy to represent a small rectal adenocarcinoma.

No patient had new, undiagnosed sites of lymphoma on PETCT outside those areas of disease established on the prechemotherapy CT.

### 3.1. Reduction in Radiation Dose and Scanning Time

In our patient cohort, the effective dose per unit-injected activity was 1.9E-2 mSv/MBq and 2.5E-02 mSv/MBq for adults and 10–15 for year olds, respectively, [17]. The mean (±SD) effective dose (ED) for whole-body PETCT was 14.6 (±1.9) mSv. The calculated mean (±SD) ED for a hypothetical scan limited to the sites of disease seen on the prechemotherapy CT was 10.6 (±2.2) mSv. This represents a mean % dose saving of 47.9% on the CT component of PETCT and a mean dose saving of 27.3% on the whole PETCT study. By limiting PETCT studies to sites of known disease, the number of bed positions scanned would have been reduced from a mean of 6 to 2 with a resultant mean saving of 16 minutes per patient.

## 4. Discussion

This study shows that limited PETCT has potential to be used for the assessment of residual disease in lymphoma without compromise to the accuracy of the investigation. It has dual advantages of reducing radiation exposure to the patient and shortening scanning time.

The risk of developing a radiation-induced cancer has been estimated at 5% per Sv in the general population but is higher in younger patients [19] and as high as 15% per Sv in the first decade of life [14]. This equates to an approximate risk between 0.5 and 1 in 1000 for an exposure of 10 mSv. In addition, genetic defects caused by radiation may contribute to the risk of cancer developing in descendants from these patients [20]. Lymphoma patients generally undergo multiple radiographic examinations during their patient journey leading to significant cumulative radiation doses to individuals, and PETCT can be a relatively high radiation dose examination. Our study estimated that patients received a mean effective dose of 14.6 mSv per standard PETCT scan comprising 8.3 mSv from CT and 6.3 mSv from PET. This is of concern as lymphoma often occurs in the young with a long life expectancy, and those subjected to high radiation dose have a significant chance of developing radiation-induced cancer [16].

However, in our study, limiting PETCT to sites of known disease would have led to a mean percentage reduction in radiation dose of 47% on the CT component and 27.3% on the whole PETCT study.

There are limitations to this study. The sample size in this retrospective study was relatively small, and we observed no cases of lymphomas in unexpected sites on postchemotherapy PETCT. This means that caution has to be applied when extrapolating the data to lymphoma patients in general. Ideally, we would like to compare pre- and postchemotherapy PETCTs so that true specificity and sensitivity would be determined. Prechemotherapy PETCT would also identify the CT-negative PET-positive incidental lesions so that the limited postchemotherapy PETCT would not miss the regions of interest.

Another limitation to this study was the inability of our scanner to optimise CT dose within an individual PET FOV containing the lesion. Irrespective of the location of a lesion, our scanner was configured to scan integer multiples of the 15.7 cm axial PET FOV rather than a limited section of this FOV. This represented a drawback to dose optimisation, as an entire FOV required more CT dose for attenuation correction compared with scanning a limited section of the FOV. For current scanners, similar to ours, where the CT range is defined as integer multiples of the axial FOV in the PET scanner, this may lead to increased effective dose compared with the ideal case where a limited FOV may be imaged. Clearly in the future, this dose issue will be optimized for scanners developed with continuous bed motion in the PET acquisition [21] and generally for noncongruent imaging ranges in PET and CT scans.

An incidental small rectal adenocarcinoma was detected in a 55-year-old man. Agress and cooper reviewed 1750 patients whose PET scans revealed 30 unexpected histopathologically confirmed malignant or premalignant tumours. This is not an inconsiderable number, but the mean age of these patients was 69 years (range 46–87) [20]. We therefore speculate that in younger patients the risk of missing a second pathology on a limited PETCT may well not be outweighed by the extra radiation dose required. The optimal cut-off age has yet to be determined. 

The estimation of change in radiation dosage is related to the number of sites of disease. Estimated mean reductions in exposure and imaging time are based on a cohort in which 60% of the patients had disease limited to one site and 90% had disease limited to 2 sites. These results would be different with a different population of lymphoma patients. Dose calculations were based on disease sites identified on prechemotherapy CT. PETCT is more sensitive for detecting lymphoma, and so it would be expected that in some patients there would be sites of disease that would not have been identified on the prechemotherapy CT. In our study, no sites of lymphoma were detected on PETCT that had not already been established on the prechemotherapy CT. There are two possible reasons for this. It may be that by chance all sites of lymphoma were detected on PETCT that had already been established on the prechemotherapy CT. The more likely reason is that there were foci of disease not identified on the prechemotherapy CT, but these were also not recognised on postchemotherapy PETCT because both occult and visible diseases on CT had been treated; a differential response is rare in lymphoma.

Nevertheless, despite these limitations, our study highlights the need to consider innovative PETCT protocols in patients with lymphoma. This is especially important with postchemotherapy studies as increasing numbers of lymphoma patients have baseline pretreatment PETCT which should accurately delineate extent of lymphoma and identify any incidental second pathology. Prospective studies, with modified PETCT protocols to reduce radiation exposure, are recommended. A further advantage of a limited PETCT is a reduction in scanning time. This could potentially translate into allowing more patients to be scanned and easing daily scheduling of patients.

## 5. Conclusion

This study suggests that response to chemotherapy in younger lymphoma patients is feasible using limited ^18^F-FDG PETCT on sites of known disease, rather than performing full (“skull base to pubic symphysis”) scans. This limited ^18^F-FDG PETCT would lead to an estimated mean reduction to radiation dose of 4 mSv (27.3%) per scan with little chance of missing significant pathology. Using limited ^18^F-FDG PETCT would also allow quicker scan times and make more efficient use of scanner resources.

## Figures and Tables

**Table 1:** Sites of disease at presentation on prechemotherapy CT. For the disease sites, the “neck” includes supraclavicular fossae, and the “chest” includes axillae.

Number of disease sites	Number of patients	Sites of disease on prechemotherapy CT	
1	60	Neck	6
		Chest	33
		Abdomen	19
		Pelvis	2

2	30	Neck and chest	15
		Chest and abdomen	11
		Other	4

3	9	Neck, chest, abdomen	5
		Chest, abdomen, pelvis	3
		Neck, chest, pelvis	1

4	1	Neck, chest, abdomen, and pelvis	1
